# RNAi-mediated knockdown of the voltage gated sodium ion channel TcNa_v_ causes mortality in *Tribolium castaneum*

**DOI:** 10.1038/srep29301

**Published:** 2016-07-14

**Authors:** Hesham M. Abd El Halim, Baida M. H. Alshukri, Munawar S. Ahmad, Erich Y. T. Nakasu, Mohammed H. Awwad, Elham M. Salama, Angharad M. R. Gatehouse, Martin G. Edwards

**Affiliations:** 1 Newcastle Institute for Research on Environment and Sustainability, School of Biology, Newcastle University, Newcastle Upon Tyne NE1 7RU, UK; 2 Entomology Department, Faculty of Science, Benha University, Benha, Egypt; 3 Department of Zoology, University of, Swabi, KPK, Pakistan; 4 Zoology Department, Faculty of Science, Benha University, Benha, Egypt

## Abstract

The voltage-gated sodium ion channel (VGSC) belongs to the largest superfamily of ion channels. Since VGSCs play key roles in physiological processes they are major targets for effective insecticides. RNA interference (RNAi) is widely used to analyse gene function, but recently, it has shown potential to contribute to novel strategies for selectively controlling agricultural insect pests. The current study evaluates the delivery of dsRNA targeted to the sodium ion channel paralytic A (TcNa_v_) gene in *Tribolium castaneum* as a viable means of controlling this insect pest. Delivery of TcNa_v_ dsRNA caused severe developmental arrest with larval mortalities up to 73% post injection of dsRNA. Injected larvae showed significant (p < 0.05) knockdown in gene expression between 30–60%. Expression was also significantly (p < 0.05) reduced in pupae following injection causing 30% and 42% knockdown for early and late pupal stages, respectively. Oral delivery of dsRNA caused dose-dependant mortalities of between 19 and 51.34%; this was accompanied by significant (p < 0.05) knockdown in gene expression following 3 days of continuous feeding. The majority of larvae injected with, or fed, dsRNA died during the final larval stage prior to pupation. This work provides evidence of a viable RNAi-based strategy for insect control.

Insect pest control in agriculture is predominantly based on the use of synthetic chemical pesticides^1^,^2^,^3^. Despite their effectiveness at controlling pest insects, there is a real need to develop alternative approaches with lower environmental and non-target impacts^4^.

Current insecticides most commonly target components of the insect nervous system^3^, often targeting the ion channels responsible for perpetuating the action potential along neurons and the enzymes of the synaptic cleft responsible for the degradation of neurotransmitters. Of these, the voltage-gated sodium channel (VGSC) is the primary target of pyrethroids^5^,^6^,^7^. VGSCs are part of a super family of ion channels that includes the voltage-gated potassium channel, the voltage-gated calcium channel, TRP-related channels and cyclic nucleotide gated channels^8^. The correct functioning of these channels is essential for normal transmission of nerve impulse and any inhibition of the action potential as a result of pesticide binding will lead to paralysis and eventual death^9^. Insect VGSCs were first cloned in the late 1980s from *Drosophila melanogaster*^10^. Although initially it was believed that *D. melanogaster* possessed two distinct isoforms of sodium ion channels, the DSC-type and the para-type, Zhou *et al*.^11^ latterly showed DSC1 to be a Ca^2+^-selective cation channel. Thus, it is the para-type that are involved in the nervous system, acting as voltage-gated sodium transporters^12^,^13^,^14^,^15^,^16^. Whilst chemicals such as pyrethroids are very effective at killing insect pests they are neither targeted, nor impervious to loss of function from evolution of insect resistance^17^. The lack of specificity of pyrethroids is partly a consequence of the VGSCs being highly conserved, not only across different insect orders but across animal species^18^. Whilst this confers broad spectrum effects of insecticidal molecules targeting these particular ion channels, it results in limited specificity and, of more concern, non-target effects. Thus there is a need to develop novel insecticides which are of comparable efficacy but demonstrate higher specificity. The use of a molecular approach such as RNA interference provides a platform enabling a highly specific and targeted strategy to insect control.

RNA interference (RNAi) represents a unique form of post-transcriptional gene silencing (PTGS); it is a recognized cellular mechanism for defence against viral invasion and post-transcriptional regulation of mRNA^19^. Further, it is the specific downregulation of gene expression mediated by an artificial double-stranded RNA (dsRNA) molecule where one strand of the dsRNA corresponds to part or all of a specific gene transcript^20^. This sequence-based interaction confers an extremely high level of specificity. Although first described in plant and animal viruses, this evolutionarily conserved phenomenon of RNA silencing has now been observed in a range of organisms from *Neurospora* (an ascomycete fungus) to much more complex organisms including insects and mammals^21^,^22^,^23^,^24^,^25^,^26^,^27^,^28^.

RNAi-based gene silencing thus has the potential to represent a novel insecticide technology, since it is theoretically possible to protect plants against insects by down regulating the expression of essential genes in the pest^20^,^29^,^30^,^31^. Furthermore, this technology should also allow non-conserved sequences to be specifically targeted, thus conferring a high degree of specificity. The red flour beetle, *Tribolium castaneum* (Tc), is a major global storage pest of grain, legumes and cereal products both for human consumption and animal feed^32^. It has been demonstrated that *T. castaneum* is readily adaptable to all currently available classes of chemical insecticide. However, it is also particularly amenable to RNAi. In addition, there are many genetic and genomic tools available for this insect and it has become the genetic model for agriculturally important coleopteran species, representing an ideal system for the identification of novel pesticide targets^33^.

The present study demonstrates that RNA interference can be used to knockdown the expression of the *D. melanogaster* DmNa_v_1 homologue in *T. castaneum*, resulting in high insect mortality. The results provide proof of concept for this approach to be used in the sustainable control of insect pests, since this strategy is both highly specific and effective towards the targeted species, and is therefore unlikely to effect beneficial insects such as pollinators and those involved in biological control, as there is no homology between the dsRNA fragment and off target sequences.

## Materials and Methods

### Insects

A culture of *Tribolium castaneum* was obtained from Blades Biological Ltd, Kent TN8 7DX and reared at 30 °C, 16:8 (L:D) on organic whole flour supplemented with 5% brewer’s yeast. Flour was replaced every 2–4 weeks.

### Design of dsRNA

Selection of the target sequence used in the present study was made using the latest version of the E-RNAi web tool (http:// www.dkfz.de/signaling/e-rnai3//)^34^,^35^. Output from E-RNAi selected a region of TC004126 transcript that had no similarities with other transcripts or low-complexity regions in the *T. castaneum* genome. The same process was employed to select a region of the kanamycin resistance gene (nptII), JN638547 (synthetic construct) from the cloning vector pSC-A-amp/kan (Stratagene) to be used as a control to assess the effect of injecting and feeding target-less dsRNA.

### Total RNA isolation and cDNA synthesis

Total RNA was isolated from 4^th^ instar larvae using TRIzol^®^ Plus RNA Purification Kit (Ambion, TRI reagent, #12183-555) following the manufacturer’s protocol. RNA integrity was evaluated on 1.5% agarose gels as described in Sambrook and Russell^36^, and quantified spectrophotometrically (NanoDrop, Labtech, ND-1000). cDNA synthesis was performed by reverse transcribing RNA using the i-Script™ reverse transcription supermix for RT-qPCR kit (BIO-RAD, 170-8841); 1000 ng of the extracted total RNA was used per each reaction.

### Synthesis of dsRNA molecules

PCR reactions were performed in a thermal cycler (Applied Biosystems, GeneAmp PCR system 2700), in 50 μl reaction volumes using (Thermo scientific PCR master mix (2x), #K0171). The final concentration of reagents used for the majority of PCRs was as follows: dNTP 0.4 mM, Magnesium Chloride 4 mM, primer concentrations 0.2 μM (Table 1), Taq DNA polymerase, 1.25 Units/reaction. The basic cycling parameters used were as follows: Denaturation step at 95 °C for 3 minutes, followed by a cycle of denaturation at 95 °C for 30 s, annealing at 57 °C for 30 s and elongation step at 72 °C for 15 s. This cycle was repeated 35 times followed by a final elongation cycle of 10 minutes. Following electrophoresis, amplified bands were gel purified using QIAquick MinElute Gel Extraction kit (Qiagen, #28604), cloned into StrataClone vector pSC-A-amp/kan (Stratagene, #240205) and then transformed into StrataClone SoloPack competent cells. Recombinant plasmids were sequenced to verify the cloned insert. The dsRNA was synthesized using replicator RNAi kit (Thermo Scientific, #F-610) and stored at −80 °C prior to injection.

### Delivery of the double-stranded RNA to larvae and pupae

Larvae and pupae (reared on organic whole wheat flour as above) were injected as described by Tomoyasu and Denell^37^, using a dissecting stereomicroscope. The dsRNA was injected into the dorsal side between the 1^st^ and 2^nd^ abdominal segments of 6^th^ (final) instar larvae, and laterally between the 2^nd^ and 3^rd^ abdominal segments for pupal stages, using a NanojectII™ injector (Drummond Scientific Company). Three biological replicates (5 larvae or pupae/replicate) were injected from one dsRNA preparation (780 ng/μl in RNAs free water, equivalent to (53.6, 106.7, 160 ng/larva and 160 ng/pupa); 48 h post injection gene knockdown was measured by RT-qPCR using gene-specific primers. For the longer-term injection bioassays, 6^th^ instar larvae (3 biological replicates, 15 larvae/rep) for each dose (53.6, 106.7, 160 ng/larva) were injected, returned to the diet and monitored on a daily basis until adult eclosion. As described previously^35^,^38^,^39^, all injected controls received RNAase free water at the same volumes as experimental conditions; a second control of dsRNA towards the microbial kanamycin resistance gene (nptII) was also included in addition to the non-injected insect group.

The dsRNA was also delivered orally via flour disks at a range of concentrations (0, 50, 100, 150 ng dsRNA/mg diet) prepared as described by Xie *et al*.^40^, in 96-wellflat-bottom well microtiter plates; one larva (6^th^ instar) was added to each well. Three biological replicates, each of 15 larvae/rep were carried out for each dose; for all feeding assays controls were used as described above. Survival of larvae was monitored until adult eclosion. To monitor gene knockdown, three biological replicate (5 larvae/rep/dose) were allowed to feed for 72 h on flour disks as described above at the same doses, after which time larvae were snap frozen in liquid nitrogen prior to assessing gene knockdown by RT-qPCR.

Since preliminary studies with different doses showed no difference in either control mortality or expression of target gene^30^, subsequent controls were only carried out at a single (highest) dose for each treatment.

### Gene expression studies with quantitative reverse transcription PCR (RT-qPCR)

cDNA, prepared as shown above, was used as the template; RT-qPCR was performed on the template in a final volume of 25 μl. Each reaction contained: 12.5 μl 2x Rotor-gene SYBR green PCR master mix solution (Qiagen Co.), forward and reverse primers (Table 1) were added in a final concentration of 0.5 mM, 9 μl nuclease free water and 1 μl of undiluted cDNA. To validate the primers, a standard curve based on a serial dilution of cDNA was carried out to determine the primer annealing efficiency, the presence of primer dimers and the production of a single PCR product. RT-qPCR conditions were as follows: 95 °C for 5 min, followed by 40 cycles of 95 °C for 15 sec, 55 °C for 30 sec and 60 °C for 15 sec. Amplifications were carried out using three biological replicates of cDNA, each one from RNA obtained from five individuals, and the mean values of three technical replicates were analyzed. The efficiency of the primers were equivalent and the relative transcript quantity was calculated according to the delta-delta Ct method^41^, with Ct values of the respective target gene compared to those of the reference gene TcRPS6 (GenBank XP_968395.1); this gene is a reliable reference gene for RT-qPCR in *T. castaneum*^42^, and hence used to normalize gene expression.

### Statistical analysis

Experimental data and qPCR results were analysed by ANOVA followed by Tukey Kramer Multiple Comparison; statistical differences are shown as different letters. Insect mortalities were analysed by Kaplan-Meyer survival analysis, SIGMAPLOT program, version 12.5, and larval mortality was corrected according to Abott’s formula^43^, with percent adult emergence calculated as previously described^44^,^45^.

## Results

### Bioinformatic analysis of targeted sequence

Homology of the TcNa_v_ dsRNA fragment designed by the E-RNAi web tool to the other 744 insect Na_v_ sequences, including representatives from important pollinator species, held by NCBI was investigated using the MegaBLAST algorithm. No homology was reported under these highly stringent conditions to any other insect species. A more relaxed BLASTn search indicated similarity between the TcNa_v_ dsRNA fragment and the Na_v_ coding sequence from a single coleopteran species, *Brassicogethes aeneus* (pollen beetle). Overall homology scores were 86% identity, total score 226 and E-value 1e-55 between the 217 base of the TcNa_v_ dsRNA fragment and the BaNa_v_ sequence.

### Temporal changes in TcNa_v_ gene expression

**Figure 1:**
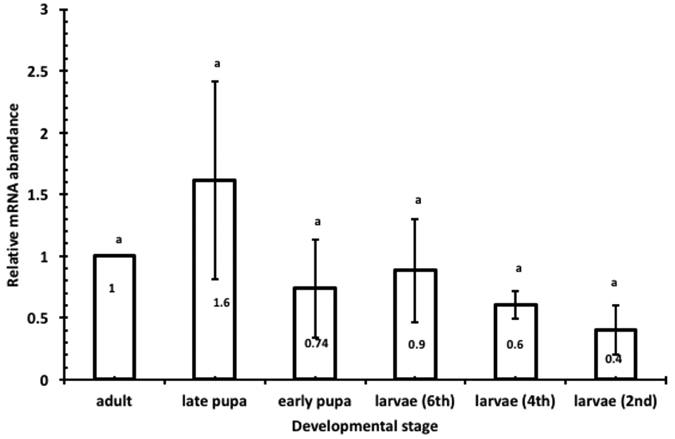
Stage-specific transcript levels in the whole body determined by qRT-PCR. Mean ± SEM of three replications are shown. Means with the same letter are not significantly different (p = 0.05; ANOVA; with Tukey Kramer Multiple Comparison).

Relative abundance of TcNa_v_ mRNA at specific life stages was estimated using qRT-PCR to ensure that subsequent studies were carried out on appropriate stages. Transcripts were detected in all life stages investigated. Relative expression of TcNa_v_ was more abundant in the late pupal stage compared to either the adult or any of the larval stages, although these differences were not significant (p > 0.05; Fig. 1). Expression in the late pupal stages was 1.6 fold greater relative to the adult stage, but lower in the larval stages, with transcript levels in 6^th^, 4^th^ and 2^nd^ instar larvae being decreased (0.9, 0.6 and 0.4-fold, respectively). Based on these relative expression levels, both pupal stages (early and late) and 6^th^ (final) instar larvae were selected as suitable stages for subsequent RNAi studies.

### Physiological and molecular response of *T. casteneum* to TcNa_v_ dsRNA

Effects on changes in expression levels of TcNa_v_ transcripts and changes in phenotype (survival and adult emergence) were investigated using both oral delivery of dsRNA and via injection.

#### Delivery of dsRNA via injection

**Figure 2:**
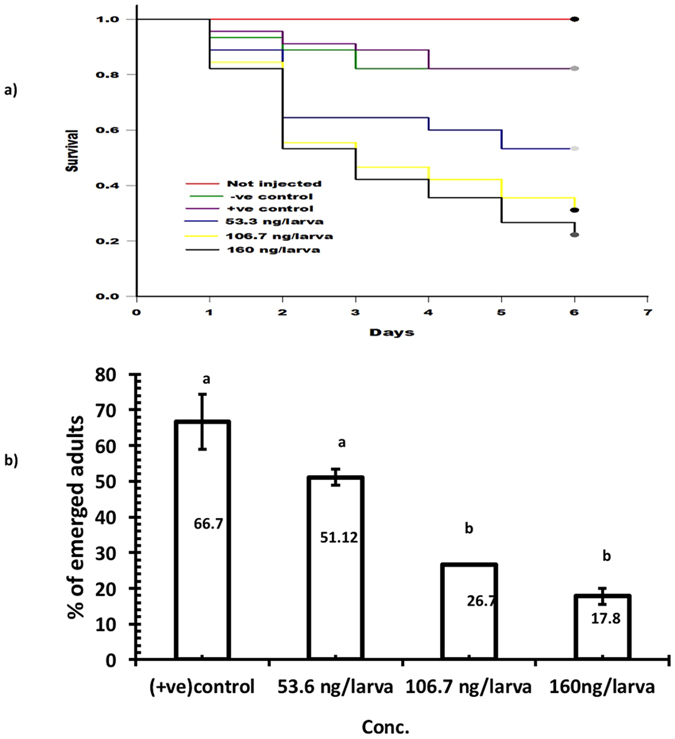
Larval survival curves and adult eclosion bioassays following injected with dsRNA. (**a**) Survival curves of larvae injected with dsRNA over 6 days, the different lines represent the survival curves for the different groups of injected larvae. (**b**) Effect on adult eclosion of dsRNA on different groups of injected larvae (p = 0.05; ANOVA; with Tukey Kramer Multiple Comparison), +ve control = larvae injected with Kanamycin resistance dsRNA, −ve control = larvae injected with RNAase free water.

The dose response effects of dsRNA directed to TcNa_v_ on survival and subsequent development into adulthood of final (6^th^) instar larvae were investigated. Larvae were monitored daily post injection until pupation, and surviving insects recorded at eclosion. Injection of dsRNA caused a significant dose dependant decrease in survival (p < 0.05), (Fig. 2a). Six days post injection, larval mortality was 35.2% ± 1.7, 62.23% ± 2.4 and 73% ± 5.9 relative to the −ve control (injected with RNase free water), when injected with dsRNA at concentrations of 53.6, 106.7 and 160 ng/larva, respectively (Fig. 2a). Over this same time period, no mortality occurred in the non-injected control larvae; the 2 control groups (RNase free water and kanamycin dsRNA) each resulted in 17.8% mortality compared to the non-injected larvae. In addition to effects on survival, injection of the dsRNA inhibited adult emergence at eclosion in a similar dose-dependent manner, with reduction in adult emergence of 51.12% ± 2.21, 26.7% ± 0 and 17.8% ± 2.22 for the increasing doses, relative to the injected control with RNAase free water (Fig. 2b). However, these differences were only significant (p < 0.05) for the two higher concentrations of dsRNA. Probit analysis determined the delivery of dsRNA via injection to have a LC_50_ of 79.89 ng/larva.

**Figure 3:**
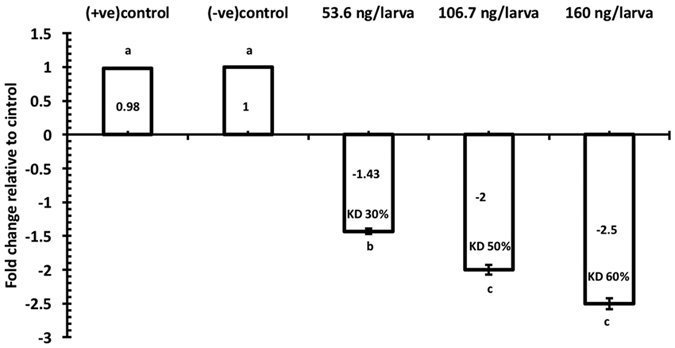
Transcript levels of TcNav mRNA after normalization with the TcRPS6 as an endogenous control 48 h post larval injection. Mean ± SEM of three replications are shown. Means with the different letters are significantly different (p = 0.05; ANOVA; with Tukey Kramer Multiple Comparison). Knockdown of transcript level is indicated for each dose (KD%) with respect to the control group, +ve control = larvae injected with Kanamycin resistance dsRNA, −ve control = larvae injected with RNAase free water.

**Figure 4:**
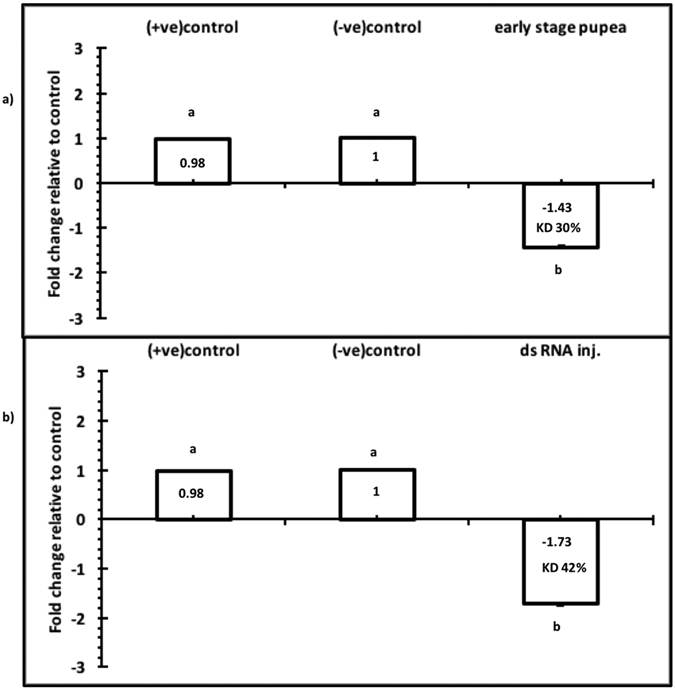
Transcript levels of TcNav mRNA after normalization with the TcRPS6 as an endogenous control 48 h post pupal injection. (**a**) Early stage pupae, (**b**) Late stage pupae. Mean ± SEM of three replications are shown. Means with the different letters are significantly different (p = 0.05; ANOVA; with Tukey Kramer Multiple Comparison). Knockdown of transcript level is indicated for each dose (KD%) with respect to the control group, +ve control = larvae injected with Kanamycin resistance dsRNA, −ve control = larvae injected with RNAase free water.

Knockdown of TcNa_v_ expression following injection of dsRNA was demonstrated using RT-qPCR (Fig. 3). Analysis showed a direct correlation between the amounts of dsRNA injected with a subsequent decrease in the abundance of TcNa_v_ mRNA transcript. Gene knockdown at 48 h post injection of 6^th^ instar larvae was shown to be significant (p < 0.05) between the different treatments, with expression of TcNa_v_ being reduced 1.43, 2.0 and 2.5-fold in response to increasing amounts of dsRNA corresponding to 53.6, 106.7 and 160 ng/larva, respectively, relative to larvae injected with RNase free water. Furthermore, qPCR analysis revealed that mRNA transcript levels in early stage and late stage pupae injected with 160 ng/pupae dsRNA were also significantly (p < 0.05) down regulated at 48 h post injection, with mRNA levels for TcNa_v_ being 1.43 and 1.73 fold lower, respectively (Fig. 4a,b) compared to their appropriate controls, which were injected with RNase free water. The differences between the two pupal stages were not significant (α = 0.05). The injection of naked off target dsRNA into either larvae or pupae did not affect expression of Na_v_ in *T. castaneum*. This dsRNA molecule designed towards a 468 bp fragment of the microbial kanamycin resistance gene (nptII) was synthesised and injected at 160 ng/larva. RT-qPCR revealed that expression of TcNa_v_ in these individuals was 0.98-fold (p > 0.05) that of the control group that were injected solely with RNase free water.

#### Oral delivery of dsRNA

**Figure 5:**
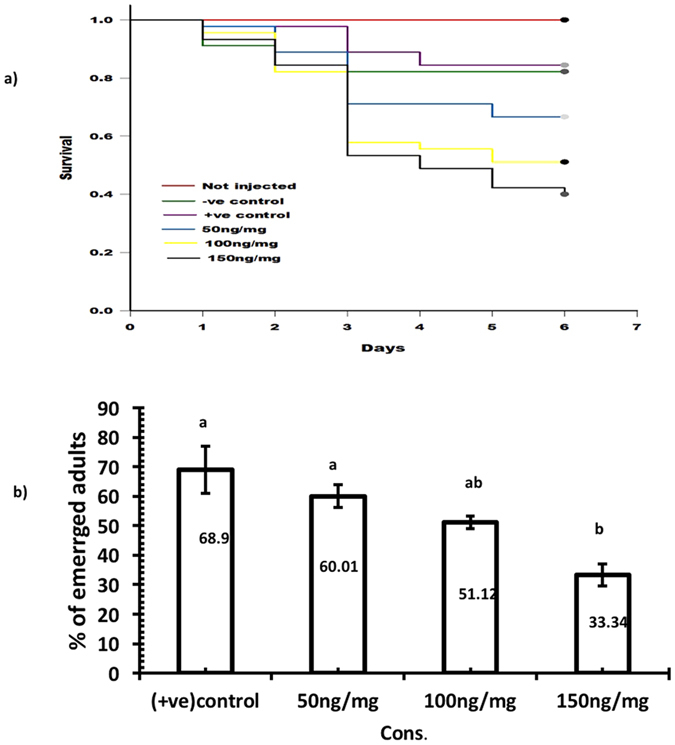
Survival curves and bioassays of larvae fed on flour disks containing TcNav dsRNA over 6 days. (**a**)Effect of dose on larval survival over time. (**b**) % of emerged adults from the different treatments above (p = 0.05; ANOVA; with Tukey Kramer Multiple Comparison), +ve control = larvae fed on Kanamycin resistance dsRNA flour disks, −ve control = larvae fed on RNAase free water flour disks.

Induction of RNA interference via oral delivery was carried out by restricted feeding of individual 6^th^ instar larvae on flour disks containing the dsRNA at a range of different concentrations (0, 50, 100 and 150 ng/mg of diet). Relative to the control diet, which fed on flour disks containing kanamycin dsRNA, feeding of the dsRNA-containing diet for 6 days resulted in a significant (p < 0.05) impact on larval survival, with larval mortality of 19% ± 6.6, 37.83% ± 0.72 and 51.34% ± 6.8 respectively, for the 3 doses, 50, 100 and 150 ng/mg (Fig. 5a). Probit analysis showed this to be a linear dose response, with an LC_50_ of 150.23 ng dsRNA/mg of diet. As with the injection of dsRNA, oral delivery also resulted in a dose dependent response. At termination of the bioassay, reduction in adult emergence from insects reaching pupation correlated with the administered dose, with the higher doses causing greater reduction in development to adulthood, with reductions of 9.9% ± 13.2, 24.35% ± 6.2 and 51.26% ± 3.8 at 50, 100 and 150 ng/mg, respectively (Fig. 5b). Although the effects of the lowest dose were not significant (p > 0.05), the two higher doses did have significant (p < 0.05) effects on adult emergence.

**Figure 6:**
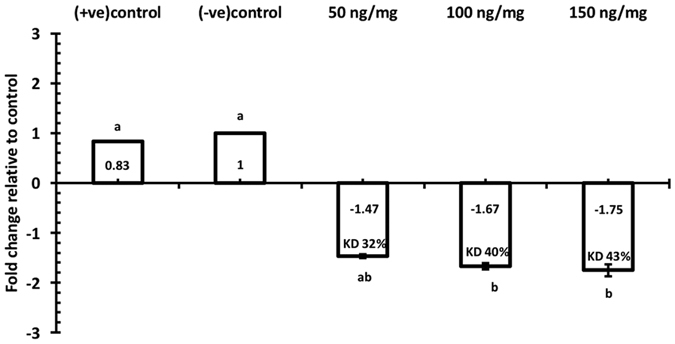
Transcript levels of TcNav mRNA after normalization with the TcRPS6 as an endogenous control 72 h post larval oral feeding. Mean ± SEM of three replications are shown. Means with the different letters are significantly different (p = 0.05; ANOVA; Tukey Kramer Multiple Comparison), +ve control = larvae fed on Kanamycin resistance dsRNA flour disks, −ve control= larvae fed on RNAase free water flour disks.

Not only did oral delivery cause a reduction in larval survival, resulting in reduced adult emergence, there was also evidence of gene knockdown. Oral delivery of dsRNA caused significant reductions in transcript levels of TcNa_v_. RT-qPCR analysis revealed that after 72 hours feeding by final (6^th^) instar larvae, transcript levels were significantly (p < 0.05) down regulated by 1.67 and 1.75-fold for the different treatments (100 and 150 ng dsRNA/mg diet) respectively, compared to the control group, which was not challenged with the TcNa_v_ dsRNA, but fed on kanamycin dsRNA flour disks (Fig. 6). However, oral delivery of dsRNA at 50 ng dsRNA/mg diet did not result in a significant decrease in gene TcNa_v_ gene expression (1.47-fold reduction, p > 0.05). Insects feeding on flour disks containing 150 ng/mg dsRNA towards the kanamycin gene did not show any significant reduction in expression of TcNa_v_ at any of the concentrations tested (0.83-fold of control, p > 0.05) compared to the group consuming control diet.

## Discussion and Conclusions

The function of the voltage-gated sodium ion channels (VGSCs) is to initiate and then propagate action potentials in the nervous system and other excitable cells^46^. The transmembrane region S4 of each domain contains 3–5 positively charged residues that are essential for the channel to respond to changes in membrane potential. In response to membrane depolarization, electrostatic interactions between positively charged residues and the depolarized cytoplasm force the S4 region towards the extracellular surface causing a conformational change in S5-S6 that opens the channel pore and allows sodium influx^47^. These ion channels are the primary target for pyrethroid insecticides which act by preventing the closure of the VGSC. The pyrethroid group of insecticides has proven to be extremely successful in the broad spectrum control of common agricultural insect pest species. However target-site resistance to these insecticides has been demonstrated in several insect orders^48^ and is based on single point mutations within the DmNa_v_ orthologues^7^.

RNA interference for suppression of specific transcripts is proving to be a powerful tool in several insect species^49^, and, in contrast to many current chemical formulations, may provide a highly species specific control strategy. Hence, such an approach has the potential to significantly reduce non-target effects, including those on beneficial insects such as parasitoid wasps and pollinators^50^,^51^,^52^. *T. castaneum* has been shown to exhibit a robust systemic RNAi response in the postembryonic stages^37^,^53^,^54^, thus it has become a model insect in which RNAi proof of concept can be demonstrated. As far as the authors are aware, this is the first study to show RNAi-induced knockdown of VGSC gene expression in insects, resulting in a dose-dependent, fatal phenotype. In the present study RNAi was induced following administration of dsRNA corresponding to the DmNa_v_ orthologue from *T. castaneum* (TcNa_v_, gene bank accession number NM_001165908.1 and TC004126 at Beetle-Base), with gene knockdown being observed following both injection into the insect haemocele and via oral uptake. Prior to delivery of the dsRNA it was first necessary to determine transcript abundances of this gene at different developmental stages to select for appropriate stages for study. Although not statistically significant, the results showed that transcript levels peaked during the late pupal stage followed by the adult and 6^th^ instar larval stages. It is perhaps not surprising to find that TcNa_v_ transcripts were more abundant in late pupal stages, as it is during this time that axons and dendrites are remodelled to generate an adult-specific arbor during metamorphosis^55^ prior to adult eclosion. These results are consistent with those reported for two acetylcholinesterase genes (TcAce1 and TcAce2), where the transcript levels were more abundant in 6-day old pupae followed by 2-day old adult *T. castaneum*^56^. Furthermore, whilst transcript levels of three genes encoding diuretic hormones were present in all developmental stages of this insect, relative expression levels were similarly dependent upon the actual development stage^57^. In contrast, analysis of the developmental stage-specific expression pattern of a lethal giant larval gene (TcLg1) in *T. castaneum* showed constitutive expression^30^.

The injection of TcNa_v_ dsRNA into the haemocele of larval *T. casteneum* induced a quantifiable RNAi effect in a dose dependent manner resulting in reduction of gene expression after 2 days. Similar observations in *T. castaneum* have been reported by Lu *et al*.^56^ where transcript levels for two acetylcholinesterase genes (TcAce1 and TcAce2) were reduced by 92.3 and 95.2%, respectively, compared to the control 4 days post injection of corresponding dsRNA fragments. Similarly, studies with the crustacean cardioactive peptide (CCAP) showed that loss of CCAP activity can be induced through RNAi with dsRNA towards TcCcapr-2. Such gene knockdown resulted in no visible abnormal phenotype but demonstrated that TcCCAPR-2 is essential for cardioacceleratory activity^58^. Furthermore, Xiao *et al*.^30^ demonstrated that *T. castaneum* larvae injected with the double stranded RNA of TcLgl (lethal giant larvae) at each of the three doses (100, 200, and 400 ng/larva) resulted in significant and dose dependant suppression of TcLgl transcripts 2 days post injection.

As with larvae, suppression of TcNa_v_ expression levels were also observed both in the early and late pupal stages, again showing consistency with those reported by^30^ where injection of 1-day old pupae with dsTcLgl, caused significant suppression of the corresponding transcript on days 2, 4, 6 and 8. Furthermore, studies demonstrated that freshly moulted pupae of *T. castaneum* injected with dsRNA led to a specific knockdown of the respective neuropeptides genes and that this dsRNA-mediated silencing caused adult loss-of-function phenotypes^59^.

The present study demonstrates, irrespective of the method of delivery, significant and severe developmental arrest (in terms of larval mortalities and reduced pupation) following knock-down of the VGSC, the severity being dependant on the dose administered. Injection of dsRNA into *Locusta migratoria* showed that locusts also have a sensitive and systemic dose-dependent RNAi response^60^. Similar responses to oral delivery of dsRNA have also been reported in *T. castaneum*^30^,^42^,^56^,^58^.

Data presented by Clemens and Elia, Oates *et al*. and Geiss *et al*.^61^,^62^,^63^ confirmed that the interferon-regulated innate immunity pathway that protects vertebrates from invasion of long dsRNA is absent in insects. It is thus feasible and preferable to deliver long dsRNAs to insects to ensure a maximal RNAi effect. Furthermore, Whyard *et al*.^50^ observed that it is possible to deliver relatively short (29–40 nt) dsRNAs designed towards insect vATPase transcripts whilst maintaining levels of induced mortality. This observation will prove advantageous if RNAi is to be developed as a viable tool in crop protection. Several *T. castaneum* genes such as heat shock protein 90, chitin synthase, the segmentation gene hairy, and a matrix metalloprotease have been showed to be down regulated by RNAi, and therefore may represent good candidate genes to target for future RNAi-based crop protection strategies. The high degree of specificity of the dsRNA molecules used in the present study towards their individual targets, as shown by the MegaBLAST homology searches, lend support for the use of this technology in crop protection. Use of this novel technology is further supported by the lack of any appreciable dsRNA uptake from the diets of higher animals. Therefore, dietary dsRNA is not anticipated to represent any hazard or risk to humans, mammals, or other vertebrates^64^,^65^. For these reasons transgenic crops expressing dsRNA are currently under investigation as a new tool for targeted and specific protection of crops from insect pests^28^,^66^,^67^,^68^,^69^,^70^.

The findings of the present study provide the first demonstration of the silencing of a voltage-gated sodium ion channel gene (TcNa_v_) via RNAi in *T. Castaneun.* It provides evidence to suggest that this gene is a feasible candidate to target for the control of this insect pest using RNAi. Furthermore, the findings presented here support the temporal expression of the genes during development, which will help in targeting the key developmental stage if this technology is to be used in crop protection.

## Additional Information

**How to cite this article**: Abd El Halim, H. M. *et al*. RNAi-mediated knockdown of the voltage gated sodium ion channel TcNa_v_ causes mortality in *Tribolium castaneum. Sci. Rep.* **6**, 29301; doi: 10.1038/srep29301 (2016).

## Figures and Tables

**Table 1:** Primers used in PCR, synthesizing dsRNA, and performing qRT-PCR.

Application Of Primers			Sequence (5′-3′)	Ampliconsize
PCR	F		AAGGCAAGGACATATTCCGA	217 bp
	R		TCAAACGTGTAGATGCCAGTG	
dsRNA synthesis	F_t7_		TAATACGACTCACTATAGGGAAGGCAAGGACATATTCCGA	217 bp
	R_phi6_		GGAAAAAAATCAAACGTGTAGATGCCAGTG	
qRT-PCR	Tc_paraA	F	TCATTCCGACTGTTGAGGGT	100 bp
		R	GATTCCCCAAAGCCCCCATC	
	Tc_RPS6	F	GAAGCAGGGTGTTCTCACGA	92 bp
		R	GTTTCCTTTCACCGTCACGC	
	Kanamycin resistance	F	TAATACGACTCACTATAGGGCATTCGCCGCCAAGTTCTTC	468 bp
		R	TAATACGACTCACTATAGGGTGCTCGACGTTGTCACTGAA	
